# Toxicity of nanoparticles_ challenges and opportunities

**DOI:** 10.1007/s42649-019-0004-6

**Published:** 2019-04-29

**Authors:** Amall Ramanathan

**Affiliations:** Department of Physics, University of Jordan, Amman, 11942 Jordan

**Keywords:** Ecotoxic, Nanoparticles, Safer design, Mechanisms of uptake, Risk assessment, Testing protocols

## Abstract

Nanomaterials (NMs) find widespread use in different industries that range from agriculture, food, medicine, pharmaceuticals, and electronics to cosmetics. It is the exceptional properties of these materials at the nanoscale, which make them successful as growth promoters, drug carriers, catalysts, filters and fillers, but a price must be paid via the potential toxity of these materials. The harmful effects of nanoparticles (NPs) to environment, human and animal health needs to be investigated and critically examined, to find appropriate solutions and lower the risks involved in the manufacture and use of these exotic materials.

The vast number and complex interaction of NM/NPs with different biological systems implies that there is no universal toxicity mechanism or assessment method. The various challenges need to be overcome and a number of research studies have been conducted during the past decade on different NMs to explore the possible mechanisms of uptake, concentrations/dosage and toxicity levels. This review article examines critically the recent reports in this field to summarize and present opportunities for safer design using case studies from published literature.

## Introduction

Nanotechnology although a desirable and innovative science, presents a large number of risks to environment and health. The very small size of NPs and the attractive surface properties (due to the large surface to volume ratio) is an important factor for many applications. However, this attractive feature may in addition, provide NP surfaces that bind and transport toxic chemical pollutants, as well as cause NPs to become toxic by generating reactive radicals. The NP size, shape, surface charge, functional groups and manufacturing methods influence its behavior along with the environmental factors (Allegri et al. 2016; Erdem et al. 2015; Korshed et al. 2016; Beranová et al. 2014; Ye et al. 2018). The specific type of organic matter or other natural particles present in sea/freshwater affects the pH and the state of aggregation or dispersion of the NPs, which plays an important role in its toxicity. NPs tend to aggregate in hard water and seawater and are greatly influenced by the surrounding environment. Toxic effects of NPs on fish and aquatic organisms even at low concentrations have been shown in literature (Lovern et al. 2007; Griffitt et al. 2008; Handy et al. 2011; Selck et al. 2016; Moore n.d.). Data also shows some manufactured NMs may interact with other contaminants, influencing their toxicity (Ye et al. 2018; Pulskamp et al. 2007).

The enormous number of NPs with their different shape, size, material, functionalities, processes of manufacture, applications etc. makes it difficult to standardize and adopt a universal measurement method for toxicity. The fate and behavior of manufactured NMs in the environment and biological matrix must be well understood; change in original structure, size, reactivity and other factors that influence the transport and behavior of NMs need to be systematically investigated as part of the toxicological studies. This is a huge challenge and at present a vast number of research studies have been conducted on various NPs to explore the possible mechanisms of uptake, concentrations/dosage and toxicity levels. Owing to the complex interactions of NPs with different biological systems there is no universal toxicity mechanism or assessment method. However, the extensive research in this area has resulted in some consensus on the following points:

- Toxicity differs with NP type and preparation and if the methodology is carefully reported, one can at least correctly interpret the whole body of measurements. Classification to NP types with common testing procedures could be made.
- The utility of behavioral assays and the need to develop more environmentally realistic bioassays. Organize existing data by relating likely exposure pathways (dermal, inhalation, ocular, ingestion) with existing measurements of important characteristics of NMs (particle number, mass, size distribution, charge) and to obtain data on toxicity mechanisms.
- The establishment of health risk assessment methodology of manufactured NMs is imperative. New types of exposure models that individually track particles through the environment while keeping track of the particle shape, surface area, and other surface characteristics as the nanoparticles are transformed or become reactive are required for modeling the behavior of NPs.
- Nanotoxicity is an emerging discipline and the importance of further research on the mechanisms and factors that increase toxicity and risk management cannot be minimized. The list of NMs is growing fast and this is a research area where continuous update of information is of vital importance.

In an attempt to put into perspective the environmental and human risks involved, the present study is a critical analysis of the toxicity of manufactured NPs based on the results of recent investigations and reviews in the field of nanotoxicity. The paper is arranged under the headings: Introduction, Toxicity of NPs, NM types and testing protocols, Case studies for safer design, and Conclusions and recommendations.

## Toxicity of nanoparticles

The ever increasing rate of NP production with its potential effects on the ecosystem and health through its intentional and unintentional release into the environment is a serious concern that needs to be addressed. NPs in healthcare, paints, cosmetics and aerosols enter the environment proportional to the use, whereas unintentional release from production facilities in the form of solid/ liquid wastes and atmospheric emissions is hard to measure and track. The NMs have the potential to contaminate land and water and enter biological systems through various pathways and cause nanostructured toxic responses. Inhalation exposure is possible with airborne NPs of size ranging from a few nanometers to several micrometers in diameter. The NPs can deposit in the respiratory system and lungs of individuals; higher deposition efficiencies have been noted for asthmatic and pulmonary disease patients as compared to healthy individuals (Kreyling et al. 2006), which may be due to the decreased clearance ability. The occupational exposure of miners and polishers to rare earth oxides can cause a severe form of pneumoconiosis (Li et al. 2014) and is discussed as a case study in section four.

**Fig. 1:**
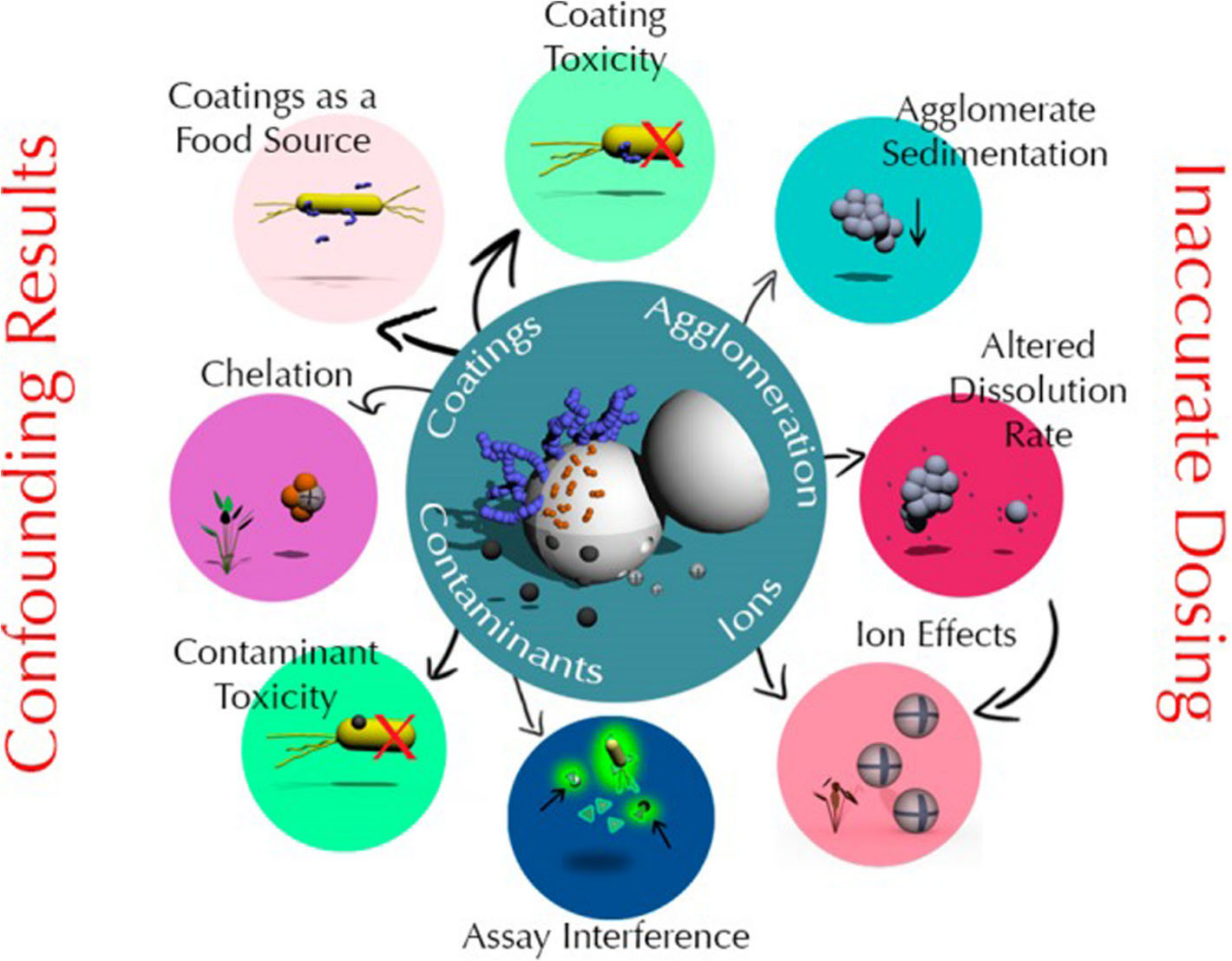
A schematic diagram showing the ways in which contaminants in the NMs, NM agglomeration, interactions between the organism and NM coating, release of dissolved ions, or interference from the NM with the assay measurement (i.e., absorbance) can potentially cause inaccurate dosing or artefacts in nano-ecotoxicology assays. Reprinted with permission from the American Chemical Society (Petersen et al. 2014)

Typical examples of metal oxides NPs like TiO_2_ and ZnO which are components of sunscreen lotions can present a potential risk upon bioaccumulation or degradation into smaller particles or transformation. Recently, researchers have come up with innovative safer design strategies for oxides of Zn and Cu (Xia et al. 2011; Sotiriou et al. 2014; Naatz et al. 2017). More details of their works are presented in section four. To what extent NPs can penetrate skin and how toxic are they, are questions that need to be answered. The testing of NP toxicity is challenging with cases of contradictory and confounding results due to the complex interactions of the NM with the organism as shown in a schematic diagram in Fig. 1. The different factors like NP coating, agglomeration, contaminants, release of ions and NM interference can be the cause of inaccurate dosing and assay results.

A useful tool and insight is how toxic are NMs at the potential concentrations they might be used and how the different factors play a role in its toxicity. The various NMs that pose a threat to humans and environment are summarized in Table 1. The NP type, shape, size, coating and functional groups all affect the behavior and toxicity of the NPs as seen from the results of recent research in this field. More details are available in the given respective references.

**Table 1:** Summary of Nanomaterial potential risks

NM	Shape/size/form/coatings	Test organism	Toxic effects/ behavioral responses	Ref.
Silca	NPs	Zebra fish	inflammation–coagulation and thrombotic effects	(Duan et al. 2018)
TiO_2_	Nano	*Daphnia magna*	no significant changes in measures of heart rate/hoping frequency	(Lovern et al. 2007)
	nanofibers	mice	significant toxic effects and more bioactive than the TiO_2_ NPs	(Allegri et al. 2016)
C_60_	suspension	Daphnia magna	significant change in heart rate	(Lovern et al. 2007)
C_60_HxC_70_Hx	suspension	Daphnia magna	hopping frequency and appendage movement to increase	(Lovern et al. 2007)
Rare earth oxides	Y_2_O_3_, La_2_O_3_, CeO_2_ etc. Mostly 18–60 nm	Humans mice	Lysosomal damage, Pulmonary inflammation and fibrosis in mouse lungs.	(Li et al. 2014)
Gadolinium based MRI agent	Gadodiamide	Patients with renal failure	Nephrogenic systemic fibrosis	(Marckmann et al. 2006)
Ag	NPs (laser generated)	5 types of human cells	low human cell toxicity weak decrease in cell survival for endothelial cells and the lung cells	(Korshed et al. 2016)
SiO_2_, TiO_2_, ZnO	NP suspensions	Gram-positive Bacillus subtilis Gram-negative *Escherichia coli*	photosensitive and harmful to varying degrees	(Adams et al. 2006)
TiO_2_	Various size and concentration NP suspensions	Gram-positive Bacillus subtilis Gram-negative Escherichia coli	induced lipid peroxidation and cellular respiration disruption	(Erdem et al. 2015)
Quantum dots	CdSe CdTe	Mice	toxicity on development of male sexual system	(Valipoor et al. 2015; Li et al. 2016)
	Carboxyl and amine surface coating	Mice and human males	lung injury and inflammation; pulmonary vascular thrombosis,	(Wu and Tang 2014; Geys et al. 2008)
Al_2_O_3_ NPs (1, 10, and 40 mg L^−1^)	Suspensions with dissolved organic matter.	unicellular green alga (*Scenedesmus obliquus*)	Disrupt cell membrane functions and trigger growth inhibition	(Ye et al. 2018)

A variety of methods of detection and identification of NPs in different environments have been used and reported in literature. Atomic force microscopy, scanning tunneling microscopy, transmission electron microscopy, confocal laser scanning microscopy, fluorescent spectroscopy, spectrophotometry, photothermal microscopy (PTM), X-ray and mass spectrometry are some of the methods used for detection and characterization (Nedosekin et al. 2015; Ostrowski et al. 2015; Hendrickson et al. 2011; Me’rian et al. 2012; Théoreta and Wilkinson 2017). Although, dark-field microscopy faces challenges on account of low sensitivity and limited axial resolution, it has found wide use in the detection, identification, and tracking of various NPs with the assistance of multispectral imaging for spectral identification of NPs (Théoreta and Wilkinson 2017; Lee and Tung 2012; Mustafa et al. 2013). Confocal fluorescence microscopy remains a powerful biological and widely used technique among the diverse imaging methods, but its drawback is the need for fluorescent NPs. Non-fluorescent NPs need to be tagged with fluorescent labels in order to use this method. With the growing evidence of label induced change in NPs, celluar structure and possible induced toxicity (Me’rian et al. 2012), using other detection methods is a wise precaution. PTM has a huge potential for absorption-based identification and quantification of non-fluorescent molecules/NPs. Recently, Nedosekin et al. (Nedosekin et al. 2015) have developed confocal PTM providing three-dimensional mapping and spectral identification of multiple chromophores and fluorophores in live cells. PTM provides a high level of sensitivity comparable to fluorescence methods with unprecedented real time detection of label free NPs in a biologically live and complex environment. This is a big plus, as the number of biomedical applications of non-fluorescent NPs for drug delivery, molecular imaging and disease diagnosis is on the rise.

## NM types and testing protocols

**Fig. 2:**
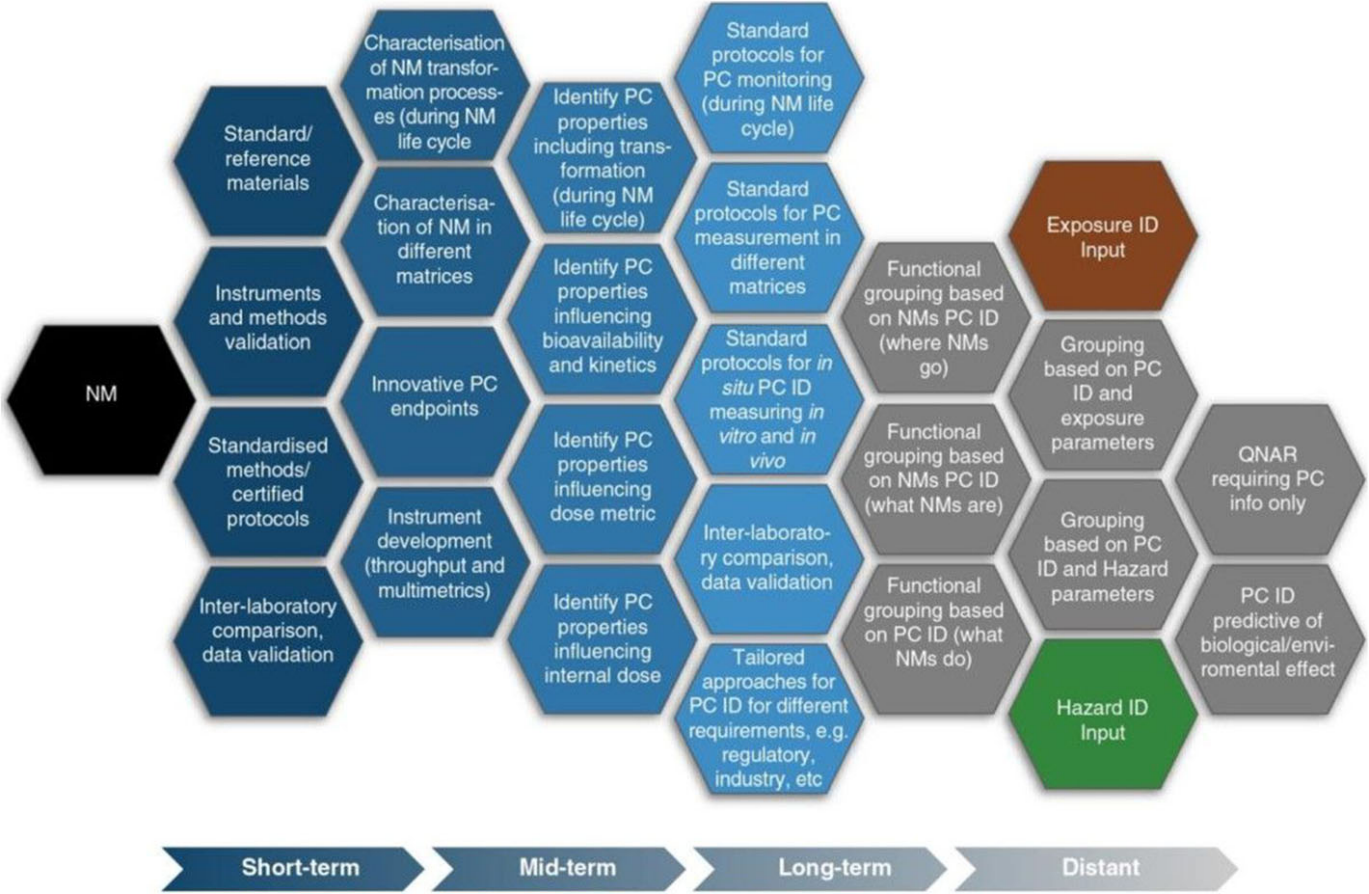
Proposed research prioritization for generating an effective PC ID to form an Intelligent Testing Strategy (Stone et al. 2014)

The extensive range of NMs in production and use today, calls for organized grouping into classes or categories with common traits. A case by case risk assessment study of NMs is not practical due to the large time and financial resources involved. A more intelligent option is to use physiochemical (PC) characterization and grouping to identify exposures and hazards and model testing procedures and safer design (Nel et al. 2013; Stone et al. 2014; Scott-Fordsmand et al. 2014; Arts et al. 2015; Godwin et al. 2015; Cobaleda-Siles et al. 2017; Kraegeloh et al. 2018) strategies. This is a complex process of establishment of tools and methods to characterize the NM during its whole life cycle and to relate the different model components to exposure and hazard inputs and come up with predictable biological/environmental risks. The work of Stone et al. (Stone et al. 2014) shows the proposed components of research priorities over different periods of time in Fig. 2. The figure depicts short, mid and long term research goals to form an intelligent testing strategy from a conclusive size, shape, composition, crystallinity, surface and other PC identification of NMs. It is then possible to maximize both functionality and safety by innovative design strategies that lower the toxicity of the NPs.

As an attempt to compartmentalize and unify testing methods the NPs can be broadly classified as metal and metal oxide, non-metal and carbon based NPs. The biggest class is the metal and metal oxide group and the most researched are Au, Ag, Cu, Zn, Si, Ti and their oxides. The effects of particle composition of Ag, Cu, Al, Ni, Co and TiO_2_ as NPs and as soluble salts on the toxicity in aquatic organisms were reported by Griffitt et al. (Griffitt et al. 2008). As a model for feeding strategies and trophic levels they used zebrafish, daphnids, and algal species. Their results show that Ag and Cu NPs cause toxicity in all organisms tested, with concentrations as low as 40 and 60 μg/L in *Daphnia pulex* adults, whereas TiO_2_ did not cause toxicity in any of the tests. The toxicity and distribution pattern of silver NPs in zebrafish model was also studied by Asharani et al. (Asharani et al. 2008), who used starch and bovine serum albumin (BSA) as capping agents for Ag NP synthesis.

A classic example of shape effect on NM toxicity is Gold (Au). Although Au NPS are generally not toxic the Au nanorods were found to be highly toxic by several research groups (Wang et al. 2008; Niidome et al. 2006; Takahashi et al. 2006; Hauck et al. 2008; Patra et al. 2007). The toxicity of Au nanorods was attributed to the surfactant, Hexadecylcetyltrimethylammonium bromide (CTAB) commonly used in the synthesis of the nanorods (Wang et al. 2008; Niidome et al. 2006). CTAB is a widely used surfactant that is used for growth assistance of the seed mediated synthesis of Au nanorods from Au NPs and facilitates anisotropic growth along the longitudinal axis and it also stabilizes the nanorods in solution. This is due to the fact that CTAB-coated nanorods are positively charged and the mutual repulsion prevents aggregation. The CTAB toxicity problem can be solved by coating with friendly and biocompatible agents like poly4-styrenesulfonic acid (PSS) (Wang et al. 2008) poly-ethylene glycol (PEG) (Niidome et al. 2006) and phosphatidylcholine (Takahashi et al. 2006). PEG is especially known for reducing the binding of non-specific molecules to surfaces. All the studies showed low/no toxicity of the treated nanorods. In fact it is possible to tune the mammalian cellular uptake of Au nanorods from very high to very low by manipulating the surface charge and functional groups of the polyelectrolyte (PE) coating schemes, as shown by Hauck et al. (Hauck et al. 2008). They used various layer-by-layer PE schemes, such as the common poly diallyldimethylammonium chloride (PDADMAC)-PSS system. In almost all cases the cell viability was found to be greater than 90%, even at very high concentrations. Their results prove the suitability of coated gold nanorods for therapeutic applications, such as thermal cancer therapy, due to the tunable cell uptake and low toxicity.

Cytotoxicity also depends on the type of cells used for testing as reported by Patra et al. (Patra et al. 2007). In their example, 33 nm citrate-capped gold nanospheres were found to be cytotoxic to a human carcinoma lung cell line at certain concentrations, but not cytotoxic to baby hamster kidney and human hepatocellular liver carcinoma cells. In the proceeding examples we saw how toxicity could be affected by a number of factors like, the method of synthesis, shape of NM and test organisms/cell lines etc. Toxicity is compellingly related to oxidative stress, gene expression, pro-inflammatory responses and cellular signaling events. In vitro and in vivo studies are very important and advance the understanding of the toxicity mechanisms to introduce a safer design and prevent unwarranted risks.

## Case studies for safer design

### Rare earth oxide nanoparticle hazard and opportunities for safer design

The possibility of occupational exposure and adverse human health effects from the mining of rare earth materials (REO) for biological and commercial use is on the increase and cases of a severe form of pneumoconiosis in miners and polishers is now common. In order to reap the benefits REO and decrease its risks by providing a safer design, the cellular interaction mechanisms were studied by Li et al. (Li et al. 2014). They investigated if engineered REO nanoparticles undergo biotransformation in THP-1 cells, a myeloid cell line that is often used as an in vitro model for studying the effects of NPs on phagocytic cells.

**Fig. 3:**
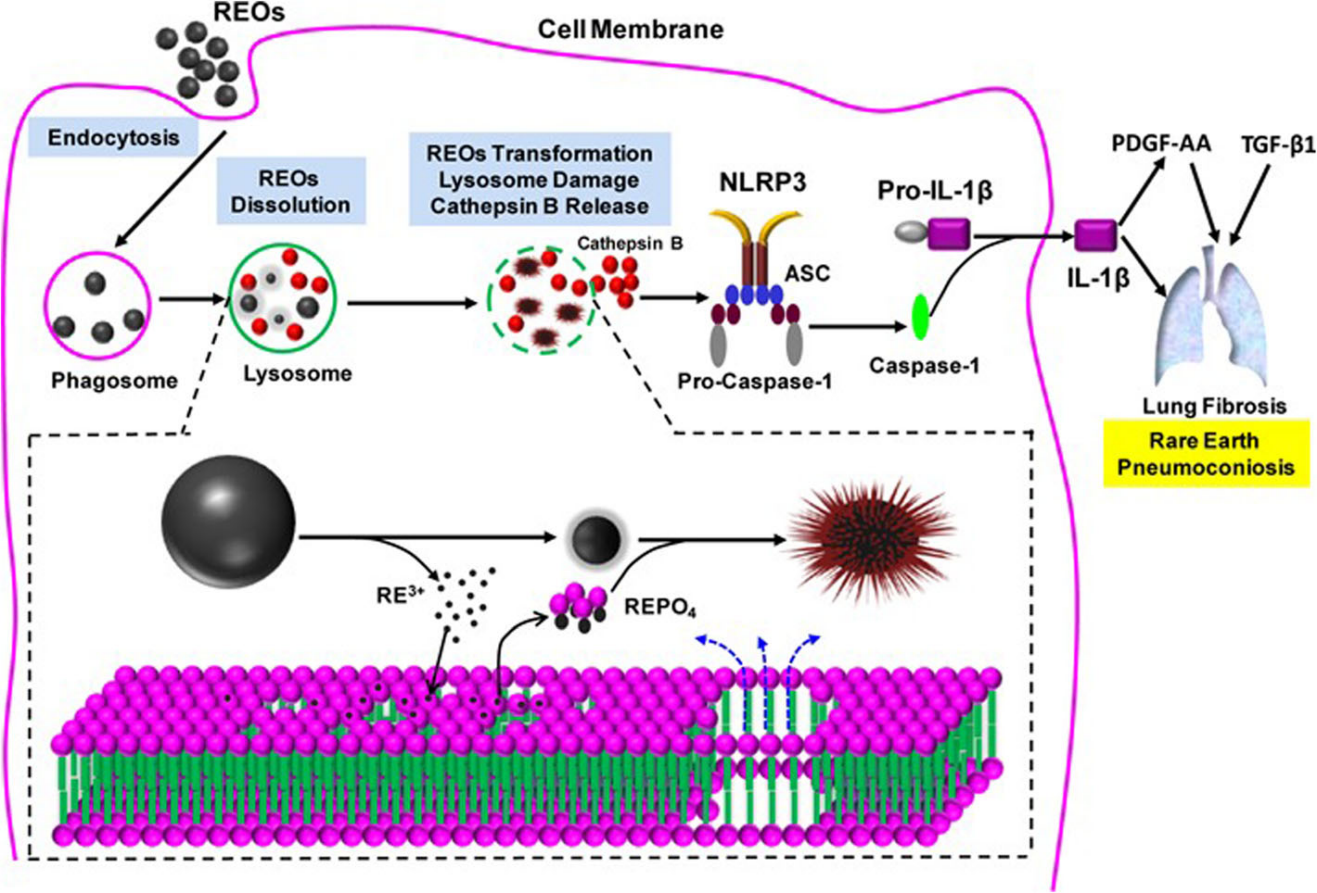
Schematics to explain the cellular mechanisms leading to the pro-fibrogenic effects of REOs. The internalization of REOs by macrophages and lysosomal biotransformation, and triggering of events culminating in pulmonary fibrosis. The inserted panel at the bottom shows that the molecular mechanism of lysosomal damage is phospholipid dephosphorylation and nucleation of REPO_4_ on the NP surface

They found that REOs induce cellular and pulmonary damage by a compartmentalized intracellular biotransformation process in lysosomes that results in urchin shaped structures. The ion shedding of REO in acidifying macrophage lysosomes leads to phosphate complexation and results in organelle damage due to stripping of phosphates from the surrounding lipid bilayer. This sets in motion a series of events as shown in Fig. 3 and causes Lung Fibrosis.

An understanding of the surface interaction mechanism of REO NPs has provided us with a safer design principle for producing rare earth nanoparticles for biological use, since, the pretreatment of REO with phosphate in a neutral pH environment stops the biological transformation and pro-fibrogenic effects and prevents lung fibrosis.

### Doping as a safer design strategy for metal oxides

Metal oxide NPs have widespread use in a number of consumer products like food, cosmetics, clothing and fuel in addition to various biomedical applications. The large amount of metal oxide NPs produced and their potential toxicity is a high health risk and requires safety evaluation. Among the metal oxides the transition metal oxides ZnO, CuO and TiO_2_ have received the most attention due to their very appealling electronic and optoelectronic properties, which allows for applications in electronics, gas sensing, solar cells, sunscreens etc. Moreover, their biocompability makes them suitable for biomedical applications like artificial medical implants and biomimic membranes etc. Unfortunately, these oxides are known to be higly toxic and it is essential to adopt a safe design strategy for their manufacture.

Xia et al. have come up with an innovative way of reducing the toxicity of ZnO NPs by Fe doping. They have shown that ZnO toxicity in mammalian cells is a result of intracellular particle dissolution and Zn^2+^ ions shedding in the tissue culture medium, which induces mitochondrial perturbation, lysosomal damage, generation of reactive oxygen species (ROS), pro inflammatory responses and cell death (Xia et al. 2008). Using a rapid throughput screening assay they have also shown that Fe-doping alters the ZnO matrix and decreases particle dissolution in tissue culture media, leading to a reduction in the above toxicological responses (George et al. 2010). In order to confirm that the doped and non-doped ZnO NPs exhibit different toxicity profiles, they compared their abilities in an in vitro screening assay that they previously developed to assess sub lethal and lethal toxicological responses in RAW 264.7 and BEAS-2B cells. More details of the screening method and results in their publication (George et al. 2010).

More recently, in order to demonstrate that the reduced toxicity of Fe doped ZnO in vitro is also reflected in vivo, they performed toxicity studies of doped and non-doped ZnO NPs using zerbra fish and rodent lung as models (Xia et al. 2011). ZnO NPs are known to interfere with zebra fish hatching and have a pro-inflammatory effect on rodent lung. Humans are also characterized by acute inflammation during occupational exposure and the use of pulmonary inflammation in rodents as a basis for tracking in vivo ZnO toxicity is therefore very relevant.

Their results on the three living models, show that Fe doped ZnO reduced the inhibitory effects of ZnO NPs on Zebra fish hatching and also reduced the pulmonary inflammation and oxidative stress in mice and rats. Kindly refer to their publication for more details of the testing procedure and elaborate discussion of results. The finding that Fe-doping leads to decreased in vitro and in vivo toxicity indicates that this is a safe design strategy for ZnO NPs, provided that the doping does not affect the electronic and commercial properties. At the level of doping required for safety, this is indeed so.

**Fig. 4:**
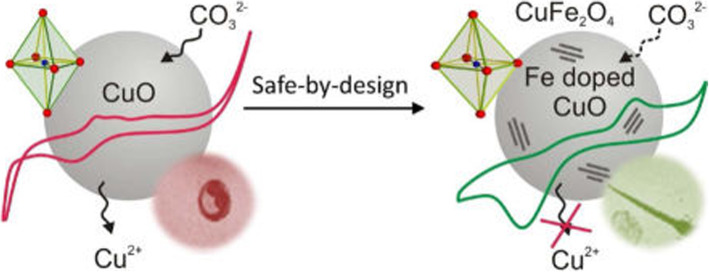
Graphical abstract (Naatz et al. 2017). Reproduced with permission from the American Chemical Society

Similar to ZnO, Fe doping on CuO also reduces its toxicity as shown by Naatz et al. (Naatz et al. 2017). CuO is widely used in industry and its dissolution has major toxic potential. So, in an attempt to reduce the dissolution by tighter binding of Cu to Fe in doped CuO, and provide a safe design they doped CuO with 1–10% of Fe in a flame spray pyrolysis reactor. Figure 4 shows a graphical abstract of their work. As shown in the figure the Fe doping causes a reduction in the dissolution and toxicity and results in a safer design for CuO NPS.

They found a significant reduction in the apical Cu-O bond length and a simultaneous increase in the planar bond length using XRD (xray diffraction), BET (Brunauer-Emmett-Teller), Raman spectroscopy, HRTEM (high resolution transmission electron microscopy), EFTEM (energy filtered transmission electron microscopy) and EELS (electron energy loss spectroscopy) for morphology and structural properties. They also performed tocicity screening and biological assessment using tissue culture cell lines and zebrafish embryos to detect the change in the hazardous effects of doped vs. non-doped particles. They demonstrated that with increased levels of doping, there was a progressive decrease in cytotoxicity in BEAS-2B and THP-1 cells, as well as an incremental decrease in the rate of hatching interference in zebrafish embryos. Pure CuO NPs exposed to THP-1 and BEAS-2B cells showed dose-dependent cytotoxicity while CuO doped with ≥6% Fe showed protective effects even at high concentration.

### Towards a safer multi-walled carbon nanotube design

Carbon nanotubes (CNT) is a NM with a wide range of applications due to its unique electronic, mechanical and thermal properties and now an indispensible material in many industries. Due to its light weight and nm size it can be easily inhaled and the occupational exposure is high. Several studies on single walled (SWCNT) and multi walled CNTs (MWCNTS) show various chronic inflammatory responses and indication of severe pulmonary and other risks (Pulskamp et al. 2007; Porter et al. 2009; Fubini et al. 2010; Poland et al. 2008; Sato et al. 2005; Yamashita et al. 2010). The toxic response of engineered NM is dependent on the physicochemical characteristics like size, shape, aspect ratio, chemical composition, stability, crystal structure, surface area, surface energy, surface charge and surface roughness (Fubini et al. 2010; Gatoo et al. 2014; Liu et al. 2013; Kostal et al. 2015). Table 2 lists some of the toxic results for various types of CNTs, with the physiochemical characterization and test cell/organism used by the research groups.

**Table 2:** List of some toxic effects of carbon nanotubes and associated physiochemical character and tests

Physiochemical character	CNT type	Test cell/organism	Toxic effects	Ref.
Contaminants	Commercial SWCNTs and MWCNTs with high content of Fe, Co, Mo and Ni	NR8383; A549	dose and time dependent increase of intracellular ROS and decrease of mitochondrial membrane potential	(Pulskamp et al. 2007)
Surface charge and modifications	acid-treated, water-soluble SWCNTs	HMMs	Are less aggregated in lysosomes and cytoplasm and cause no significant changes in cell viability or structure.	(Porter et al. 2009)
Shape	pristine SWCNTs; oxidized SWCNTs	HUVEC	functionalized and pristine SWCNTs have limited cytotoxicity	(Fubini et al. 2010)
	asbestos-like MWCNTs	Normal mice	length-dependent inflammation and formation of granulomas	(Poland et al. 2008)
	tangled CNTs; needle-like CNTs	human primary macrophages	tangled CNTs are swallowed into cells; needle-like CNTs activate secretion of IL-1R and IL-1β	(Palomaki et al. 2011)
Length	MWCNTs 500 nm to 5 μm	THP-1; rat	average length of 825 nm induce higher inflammation than those with an average length of 220 nm	(Sato et al. 2005)
	MWCNTs: length 5 to 15 μm, diameter 20-60 nm; SWCNTs: length 5 to 15 μm, diameter < 2 nm	A549; THP-1; normal mice	long and thick MWCNTs induce the strongest DNA damage similar SWCNTs caused little effect	(Yamashita et al. 2010)
	long MWCNTs 1 to 20 μm	p53+/ mice	Long MWNTs can form fibrous or rod-shaped particles of around 10–20 μm (μm) and induce mesothelioma	(Takagi et al. 2008)
Number of layers	Pristine graphene	RAW 264.7	depletion of the mitochondrial membrane potential and increase of intracellular ROS and apoptosis.	(Li et al. 2012)
	SWCNTs; MWCNTs diameters 10 to 20 nm	alveolar macrophage	SWCNTs > MWCNTs	(Jia et al. 2005)
Agglomeration	agglomerated SWCNTs; better dispersed SWCNT bundles CNT agglomerates; CNT bundles	SPC; DRG MSTO-211H	highly agglomerated SWCNTs significantly decrease the overall DNA content	(Belyanskaya et al. 2009)
			suspended CNT-bundles are less cytotoxic than asbestos, rope-like agglomerates	(Wick et al. 2007)

Considering the potential health risks of CNTs in all its different forms there is a growing demand for guidelines for safer CNT design. Surface charge plays a major role in the interactions of NPs with the biological systems (Lee et al. 2013; Bozich et al. 2014; Harper et al. 2011) and research groups have employed various ways to to reduce the NP toxic manifestations by screening or modulating the surface characteristics. One such group, Gilbertson et al. (Gilbertson et al. 2016) have established a statistical model relating surface charge and embroyonic zebrafish mortality for a safer MWCNT design. The zebrafish are a preferred in vivo vertebrate model organism and have molecular and physiological conservation with other vertebrates, particularly during embryonic development (Fako and Furgeson 2009; Rizzo et al. 2013; Usenko et al. 2007). It is a stable, rapid and sensitive biological model with many genetic parallels with humans enabling understanding and translation to potential human toxicity mechanisms. In their study the bioactivity of zebrafish was monitored upon exposure to systematically modify and completely characterise MWCNTS. The surface charge, percent surface oxygen, dispersed aggregate size and morphology, and electrochemical activity were the four physicochemical properties of MWCNTs that were considered by them. Their analysis indicted that the surface charge was the best predictor of zebrafish mortality at 24 h post fertilization (hpf). This conclusion was obtained by their use of advanced multivariate statistical methods, a model that identifies the physicochemical property that best estimates the probability of observing an adverse response. This type of statistical predictive approach has been identified as primary research objective for optimization and safety of NMs (Liu et al. 2013; Usenko et al. 2007). In the statistical model they considered seven samples with the four PC properties and the surface charge quantified as point of zero charge (PZC) values ranged from 3 to 9. The relavance of this model is therefore for carbon nanotubes with PZC properties within this range. Even with the limitation of low sample size they were able to predict and validate MWCNT surface charge as the best predictor of zebrafish mortality at 24 hpf and the identification of this property-risk relationship establishes a foundation for the development of low risk design guidelines for MWCNTs.

These case studies highlight the need for caution during the use and design of manufactured NPs to prevent human and environmental adverse impacts, as well as the importance of further research on the mechanisms and factors that increase toxicity to formulate a safe and risk free NP/NMs design.

## Conclusions and recommendations

In conclusion, the widespread use of NPs in various applications over industries ranging from electronics, medicine, pharmaceuticals, and cosmetics to agriculture and food will result in intentional/ unintentional human exposure to engineered NP/NMs via the dermal, inhalation, ingestion, and ocular pathways. Control measures that can be adopted are

i. Reduce workplace exposures by using engineering controls and personal protective equipment. Use alternative methods to assess exposure and the influence on bioavailability and toxicity.
ii. Find alternatives to the use of surfactants, templates, or other substances that are used to stabilize and control nanoparticle size and shape during synthesis which are toxic.
iii. Use green and sustainable synthesis methods of manufacture of NPs which depend on natural resources like plant extracts, microorganisms and energy saving methods in an economical and non-toxic manner (Ramanathan and Aqra n.d.; Ahmed et al. 2016; Dahoumane et al. 2016; Kitching et al. 2015; Mahdavi et al. 2013).
iv. Use standard nanoparticle measurement methods_respirable mass or number concentration and extended in vivo and/or in vitro toxicity testing with uptake, internal distribution and use of realistic risk assessment methods.
v. Comprehensive NP/NM physiochemical characterization and transformation studies in environmental and biological matrices and the consequences for bioavailability and toxicity in test organisms.
vi. Develop excellent understanding of NP property-activity/risk relation to provide safe design.

It is highly recommended to develop a framework of green nanoscience that can guide safe design, production, and application of NMs across a wide range of industries by effective characterization of the NM during the entire product lifecycle using a defined set of standardized metrics that can be consistently measured. The occupational, consumer and environmental exposure should be monitored by devices capable of detecting the physiochemical properties of the NP/NMs progress in the exposure matrix even if they are transformed or become reactive and trigger biological responses. To reap the potential benefits of products of nanotechnology, nano-safety research should be prioritized to develop an intelligent testing strategy and a set of NM risk management protocols (Nel et al. 2013; Stone et al. 2014; Scott-Fordsmand et al. 2014; Arts et al. 2015; Godwin et al. 2015; N.R.C 2012; Notter et al. 2014; Voutchkova-Kostal et al. 2012; Yang et al. 2014; Handy et al. 2012; N.N.I 2011).

## Abbreviations

BET: Brunauer-Emmett-Teller
CNT: Carbon nanotube
CTAB: Hexadecylcetyltrimethylammonium bromide
EELS: Electron energy loss spectroscopy
EFTEM: Energy filtered transmission electron microscopy
hpf: Hours post fertilization
HRTEM: High resolution transmission electron microscopy
MWCNT: Multi-walled carbon nanotube
NMs: Nanomaterials
NPs: Nanoparticles
PC: Physiochemical
PDADMAC: Poly diallyldimethylammonium chloride
PEG: Poly-ethylene glycol
PSS: Poly4-styrenesulfonic acid
PTM: Photothermal microscopy
PZC: Point of zero charge
SWCNT: Single-walled carbon nanotube
